# Prevalence of pathogens from clinical samples associated with porcine respiratory and digestive diseases in South Korea from 2021 to 2023

**DOI:** 10.3389/fvets.2025.1461935

**Published:** 2025-06-30

**Authors:** Hye-young Wang, Joong Ki Song, Seongho Shin, Hyunil Kim

**Affiliations:** ^1^Optipharm Inc., Cheongju-si, Republic of Korea; ^2^Optipharm Animal Disease Diagnostic Center, Cheongju-si, Republic of Korea

**Keywords:** prevalence, respiratory, reproductive, digestive, pathogen, multiplex real-time PCR, diagnosis

## Abstract

Respiratory and digestive diseases cause significant losses in the swine industry. The current study aimed to investigate the prevalence of viruses and bacteria associated with respiratory/reproductive and digestive diseases in pigs. Clinical samples were collected from 230 farms in South Korea between 2021 and 2023 from pigs with suspected diseases. The pigs were screened for pathogens related to respiratory/reproductive and digestive diseases via multiplex real-time polymerase chain reaction. Of the 104,128 samples, 28,281 [27.2%, 95% confidence interval (CI): 26.9%−27.4%] tested positive for pathogens. The overall prevalence of pathogens related to respiratory/reproductive and digestive diseases was 74.7% (*n* = 21,145, 95% CI: 74.2%−75.2%) and 25.3% (*n* = 7,136, 95% CI: 24.7%−25.7%), respectively. Among these pathogens, porcine reproductive and respiratory syndrome virus (PRRSV, *n* = 11,997, 56.7%, 95% CI: 56.1%−57.4%) and rotavirus (*n* = 4,430, 62.1%, 95% CI: 60.93%−63.2%) were the most prevalent. The trends in 3-year prevalence showed no significant changes, but in 2023, viral infections (e.g., PRRSV, Rotavirus, porcine epidemic diarrhea virus, etc.) decreased and bacterial infections (e.g., *Mycoplasma hyopneumoniae* (MH), *Pasteurella multocida* (PM), *Haemophilus parasuis* (HP), *Salmonella* spp., *Lawsonia intracellularis*, and *Brachyspira hyodysenteriae*) slightly increased (χ^2^ = 11.36, *P* < 0.001). An investigation of seasonal characteristics revealed that the prevalence of some respiratory pathogens such as PRRSV and HP was higher in winter than in other seasons, and the prevalence of digestive bacterial pathogens such as *Salmonella* spp., *L. intracellularis*, and *B. hyodysenteriae* was higher in summer than in other seasons. The study results, including the prevalence of viruses and bacteria, patterns of pathogen frequency, annual distribution status, and seasonal characteristics, are helpful in understanding pathogen trends in porcine respiratory/reproductive and digestive diseases.

## Introduction

Pigs serve as hosts for pathogens that cause diseases transmissible to humans. In addition, they are a source of pathogens that can cause fatal disease outbreaks in humans and animals. Respiratory and reproductive diseases in pigs can result in poor growth, reduced feed efficiency, loss of appetite, breathing difficulties, reproductive failure, and increased mortality, all of which reduce their productivity. Furthermore, they can contribute to substantial economic losses in the swine industry (1, 2).

Representative pathogens that cause respiratory and reproductive diseases include viruses such as porcine reproductive and respiratory syndrome virus (PRRSV), porcine circovirus type 2 (PCV2), Aujezsky disease virus (ADV), swine influenza virus (SIV), Japanese encephalitis virus (JEV), encephalomyocarditis virus (EMCV), porcine parvovirus (PPV), and porcine cytomegalovirus (PCMV), as well as bacteria such as *Haemophilus (Glaesserella) parasuis* (HP), *Pasteurella multocida* (PM), *Mycoplasma hyopneumoniae* (MH), and *Actinobacillus pleuropneumoniae* (APP). Porcine reproductive and respiratory syndrome virus (PRRSV), PCV2, and ADV are major pathogens of the porcine respiratory disease complex (PRDC), which causes severe lesions and respiratory immune system issues, making the host susceptible to infection caused by other respiratory pathogens (2). Bacteria are common secondary infectious pathogens in swine farms. Moreover, highly pathogenic bacteria can be a major cause of primary infection and acute mortality.

Diarrhea is a common condition causes dehydration, and mortality in pigs. Diarrheal diseases in pigs also have a significant economic impact, including high mortality rates, changes in body weight and changes in feed conversion ratios because of longer stays at farms (3). The primary pathogens of diarrhea-related digestive diseases include viruses such as rotavirus, porcine epidemic diarrhea virus (PEDV), and transmissible gastroenteritis virus (TGEV), as well as bacteria such as various species of the genus *Salmonella* (Sal) by targeting common genes (4), *Lawsonia intracellularis* (Law), *Brachyspira hyodysenteriae* (BH), and *Brachyspira pilosicoli* (BP) (5).

To date, several studies have reported on the prevalence of each pathogen causing respiratory, reproductive, and digestive diseases in various countries (1, 2, 5–10). However, a survey on the simultaneous prevalence of these pathogens has not been conducted. The current study investigated the prevalence and characteristics of viral and bacterial pathogens as well as seasonal changes in porcine diseases outbreaks in selected regions of South Korea from 2021 to 2023.

## Methods

### Sample preparation

**Figure 1:**
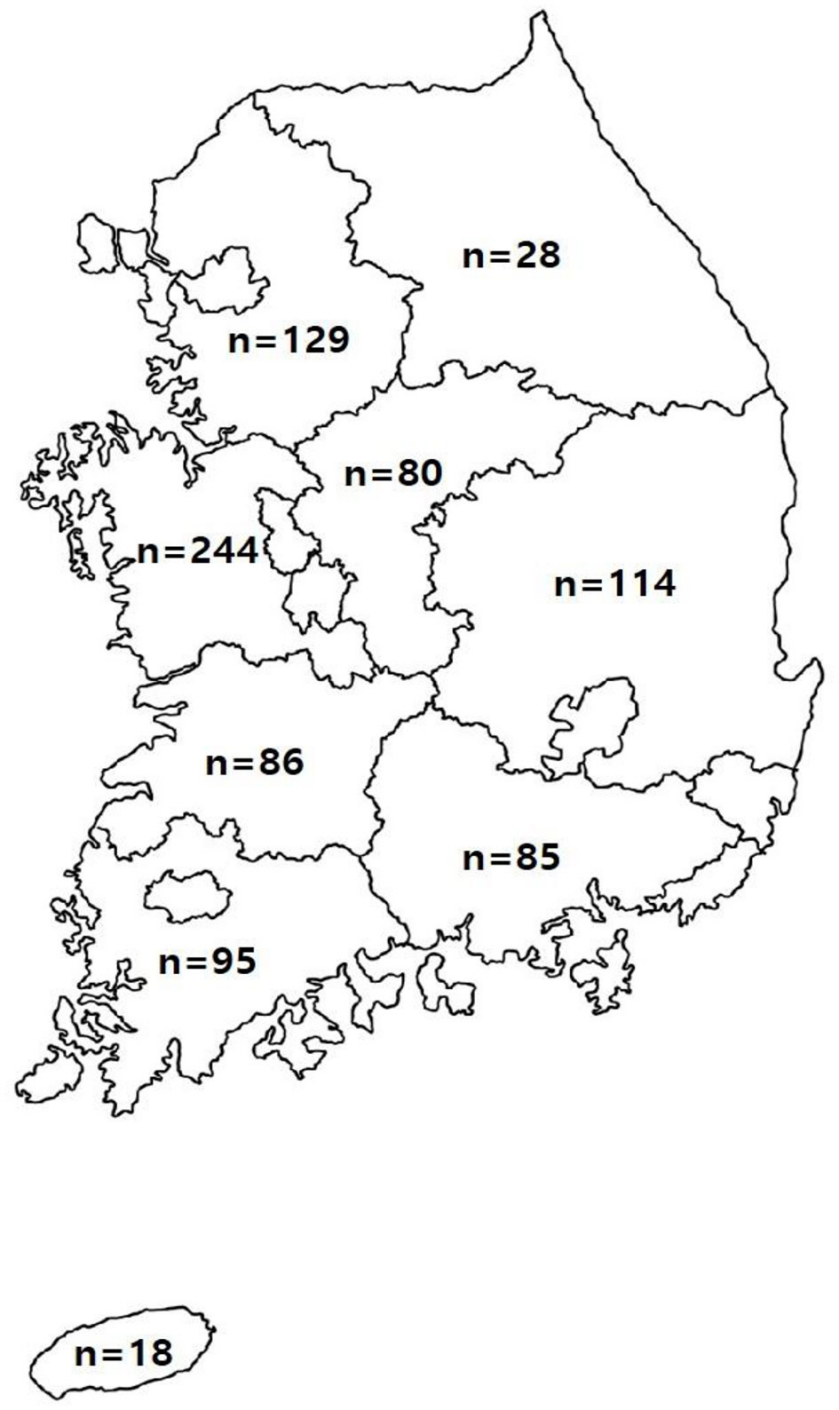
Location and number of farms requested for the testing of samples suspected of being infected with pathogens causing swine diseases in the Republic of Korea. The number of farms located in each province is indicated.

To evaluate suspected diseases, 104,128 samples were collected from 879 farms in various regions of the Republic of Korea and transferred to the Optipharm Animal Disease Diagnostic Center from 2021 to 2023 (Figure 1). All animal studies and protocols were approved by the Optipharm Animal Disease Diagnostic Center and Use Committee (ACE 2020-010) in compliance with ethical requirements. According to the manufacturer's recommendation (3, 11), samples such as blood serum, tissue, and feces collected for testing were immediately pretreated with phosphate-buffered saline, and DNA/RNA was extracted from 100 μL of the sample using a commercial automated nucleic acid extraction system (Miracle-AutoXT Automated Nucleic Acid Extraction System, Intronbio, Seongnam, Republic of Korea). To prevent cross-contamination, all samples, including pretreatment samples remaining after extraction, were individually processed and stored at −20°C. The content and purity of the extracted DNA/RNA were analyzed by measuring the absorbance at 260 and 280 nm using a spectrophotometer (Infinite^®^ 200 NanoQuant, Tecan, Switzerland).

### Multiplex real-time polymerase chain reaction assay

The multiplex real-time polymerase chain reaction (PCR) assay was performed using commercially available products for the simultaneous detection of multiple pathogens, as listed in Table 1 (Supplementary Table 1). Furthermore, multiplex real-time PCR was performed using a CFX-96 real-time PCR system (Bio-Rad, Hercules, CA, USA) for thermal cycling and fluorescence detection, according to the manufacturer's recommendation. Real-time PCR amplification was performed with a total reaction volume of 20 μL by adding 5 μL template DNA/RNA to each tube comprising eight strips mixed with Taq enzyme, buffer, and Primer & TaqMan Probe that were labeled with fluorophores (FAM/HEX-BHQ1, CalRed610/Cy5-BHQ2). Positive (plasmid DNA) and negative controls comprising molecular-grade (DNAse/RNAse-free) water (ultrapure water; Welgene, Gyeongsan, Republic of Korea) without template DNA/RNA were included in each assay. The assay was performed under the following conditions: For DNA, an initial denaturation at 95°C for 3 min, 10 cycles for 3 s at 95°C and 30 s at 60°C, and 40 cycles for 3 s at 95°C and 30 s at 55°C. For RNA, reverse transcription was performed at 50°C for 2 min, an initial denaturation at 95°C for 2 min, 10 cycles for 3 s at 95°C and 30 s at 60°C, and 40 cycles for 3 s at 95°C and 30 s at 55°C. Each sample was tested in duplicate by running the PCR cycle twice, and a positive result was obtained if the *C*_*T*_ value was < 35.

### Statistical analysis

All statistical analyses were performed using Prism 5 software (GraphPad, La Jolla, CA) and Statistical Package for the Social Sciences software version 21.0 (IBM, Armonk, NY, USA). The prevalence of each pathogen was calculated using the chi-square test (χ^2^), along with the corresponding *P* value and the 95% confidence interval (CI) for predictive ability. A *P* value < 0.05 was considered statistically significant.

## Results and discussion

### Overall prevalence of pathogens isolated from 2021 to 2023

**Figure 2:**
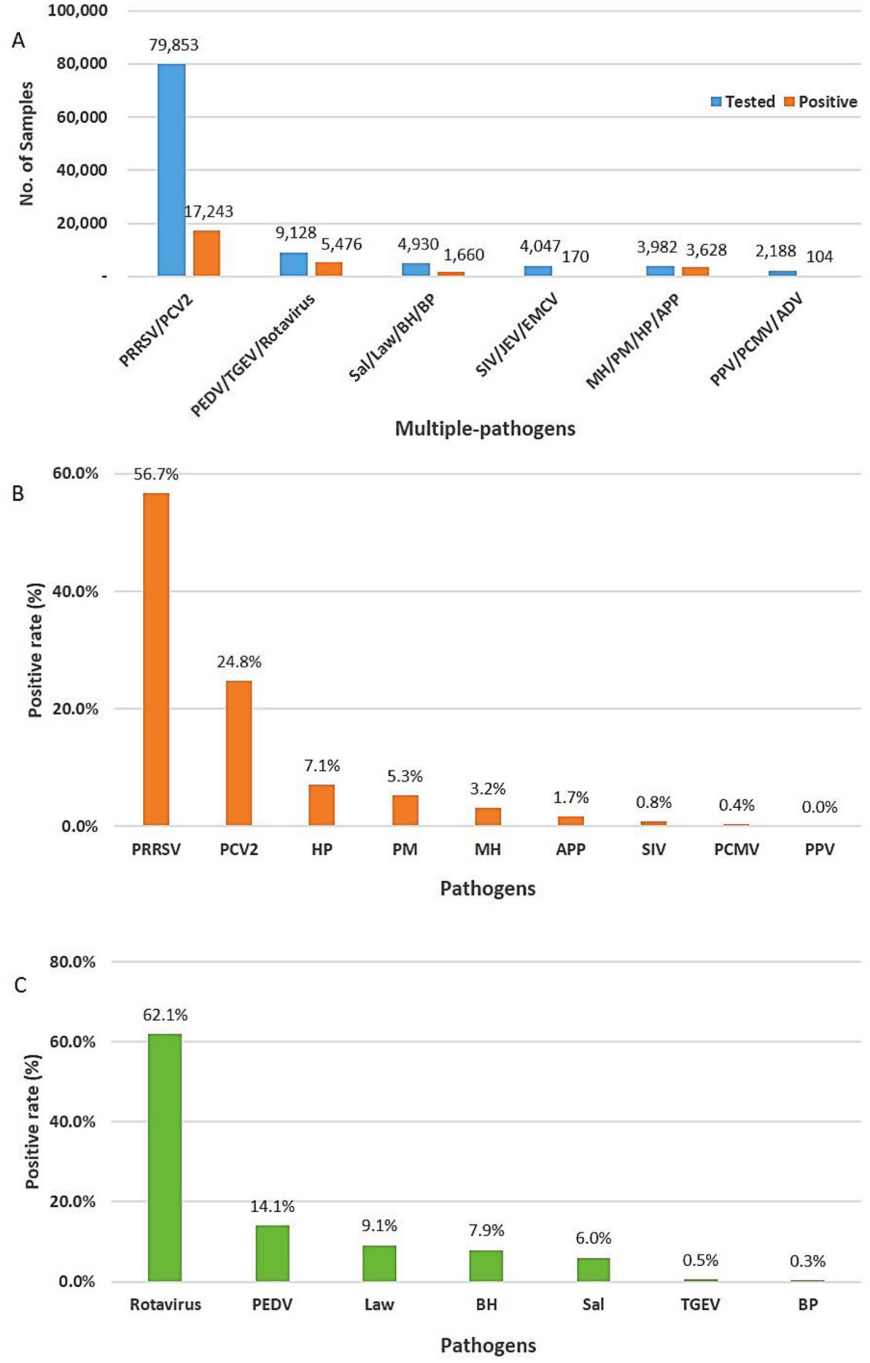
Positivity rate of different viruses and bacteria. **(A)** Total number of tests and positive rate for multiple pathogens using multiplex real-time PCR assay. **(B)** Respiratory and reproductive pathogens. **(C)** Digestive pathogens.

In total, 104,128 various types of samples, such as blood, serum, organs, swabs, and feces, were collected from different regions of South Korea from 2021 to 2023. Using multiplex real-time PCR comprising multiple pathogen combinations, 28,281 (27.2%, 95% CI: 26.9%−27.4%) of 104,128 samples tested positive for different pathogens, including highly pathogenic porcine reproductive and respiratory syndrome virus (HP-PRRSV)/PCV2 (*n* = 17,243, 60.9%, 95% CI: 60.4%−61.5%), PEDV/TGEV/rotavirus (*n* = 5,476, 19.4%, 95% CI: 18.9%−19.8%), Sal/Law/BH/BP (*n* = 1,660, 5.9%, 95% CI: 5.6%−6.1%), SIV/JEV/EMCV (*n* = 170, 0.6%, 95% CI: 0.5%−0.7%), MH/PM/HP/APP (*n* = 3,628, 12.8%, 95% CI: 12.4%−13.2%), and PPV/PCMV/ADV (*n* = 104, 0.4%, 95% CI: 0.3%−0.4%; Figure 2).

### Prevalence of respiratory and reproductive pathogens

In a total of 28,281 positive samples, the prevalence of pathogens related to respiratory and reproductive diseases was 74.7% (*n* = 21,145, 95% CI: 74.2%−75.2%). Of the identified pathogens, approximately 82.7% (*n* = 17,517, 95% CI: 82.3%−83.3%) and 17.3% (*n* = 3,623, 95% CI: 16.7%−17.7%) were viruses and bacteria, respectively. Porcine reproductive and respiratory syndrome virus (PRRSV, *n* = 11,997, 56.7%, 95% CI: 56.1%−57.4%) was the highest, followed by PCV2 (*n* = 5,246, 24.9%, 95% CI: 24.2%−25.4%), HP (*n* = 1,497, 7.1%, 95% CI: 6.7%−7.4%), PM (*n* = 1,105, 5.3%, 95% CI: 4.9%−5.5%), MH (*n* = 664, 3.2%, 95% CI: 2.9%−3.3%), and APP (*n* = 362, 1.7%, 95% CI: 1.5%−1.9%; Figure 2). Porcine reproductive and respiratory syndrome virus (PRRSV) remains a highly pathogenic disease that threatens the swine industry in South Korea. Eradicating PCV2 from previously contaminated farms is challenging, and the damage is often exacerbated by secondary or concurrent infections with PCV2 (12). They can be transmitted via various routes, such as contact with contaminated urine and feces, contact between individuals, and airborne transmission. Hence, continuous circulating infection occurs in infected farms (13, 14). The prevalence of PRRSV and PCV2 in this study was slightly higher than that reported in previous study (41.16% vs. 21.58%) in China (2), and the average annual co-infection of PRRSV and PCV2 in this study (*n* = 2,013, 16.7%, 95% CI: 14.2%−18.3%; Supplementary Table 2) were lower than those reported in previous studies (*n* = 1,307, 24.94%). The prevalence of SIV (*n* = 170, 0.8%, 95% CI: 0.6%−0.9%), PCMV (*n* = 97, 0.3%, 95% CI: 0.3%−0.5%), and PPV (*n* = 7, 0%) was relatively lower than that of other respiratory and reproductive pathogens. This study did not identify ADV, a major pathogen associated with PRDC (2), JEV, and EMCV, which are linked to reproductive failure. These results are consistent with those of reports indicating that ADV has not been observed in South Korea since 2010 and that vaccination has been discontinued after 2013 (1, 15). The overall frequency of bacteria tested was significantly lower than that of viruses. HP, PM, and MH, which are secondary pathogens causing PRDC, remain major respiratory pathogens in South Korea, and especially virulent strains can cause major symptoms (2).

**Figure 3:**
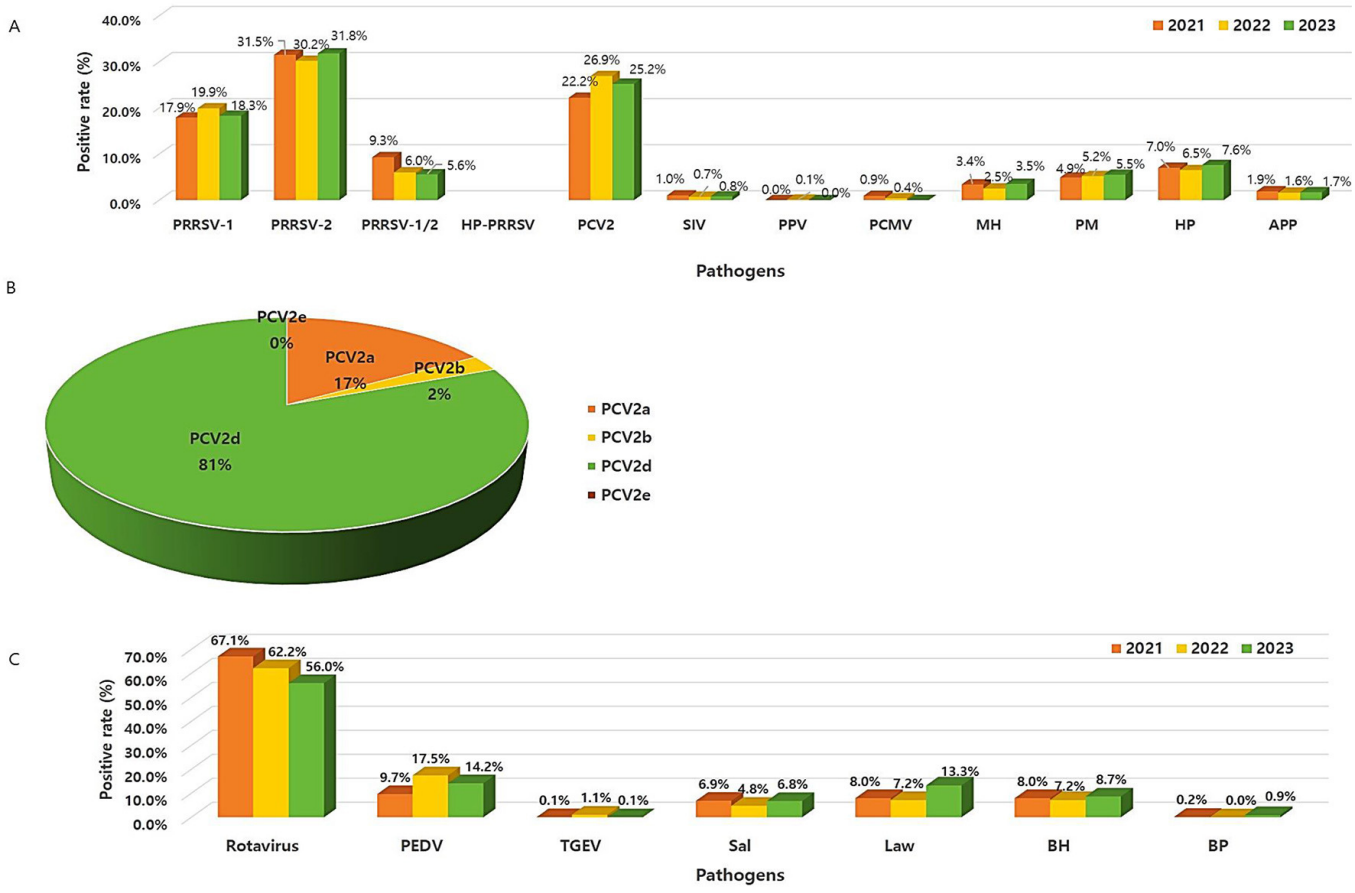
Frequency pattern of different viruses and bacteria from 2021 to 2023. **(A)** Respiratory and reproductive pathogens. **(B)** Prevalence of the PCV2 genotypes. **(C)** Digestive pathogens.

We investigated trends in the prevalence of respiratory pathogens over the three years from 2021 to 2023 (Figure 3A). First, PRRSV was divided into PRRSV-1 (type 1, European) and PRRSV-2 (type 2, North American). The average 3-year prevalence of PRRSV-1 and PRRSV-2 was ~18.7% (95% CI: 18.3%−19.1%) and 31.2% (95% CI: 30.8%−31.5%), respectively. The co-infection of PRRSV-1 and PRRSV-2 was 7% (95% CI: 6.2%−7.8%) (Supplementary Table 2). The annual prevalence patterns for most of these was similar, and the detection of PRRSV-2 was more than 1.5 times higher than that of PRRSV-1. This result was similar to that of a previous study (12.2% vs. 7.6%) (16). Highly pathogenic porcine reproductive and respiratory syndrome virus (HP-PRRSV) is a highly pathogenic/virulent variant of PRRSV and is characterized by high morbidity and mortality in pigs of all ages. Highly pathogenic porcine reproductive and respiratory syndrome virus (HP-PRRSV) initially emerged in China and Vietnam in 2006 and later in Southeast Asian countries including Malaysia, Laos, the Philippines, and Cambodia (6). Thus, the prevalence of HP-PRRSV was assessed starting in 2021, and no pathogen has been found to date. Second, the 3-year prevalence trend of PCV2 was inconsistent, with the average prevalence being approximately 24.8% (95% CI: 23.88%−25.7%). In addition, PCV2 genotyping was performed on some samples confirmed to be PCV2-positive (Figure 3B). Hence, PCV2d had the highest prevalence at 81% (*n* = 2,665, 95% CI: 75%−85.3%), followed by PCV2a at 17.4% (*n* = 547, 95% CI: 12.8%−21.9%) and PCV2b at 2.5% (*n* = 80, 95% CI: 2%−3.1%). PCV2e was not detected in this study. Compared with PCV2a and PCV2b, PCV2d is known to cause more severe clinical symptoms in pigs (17). Currently, PCV2 vaccines based on PCV2a are considered effective in reducing PCV2d viremia, and these vaccines can effectively facilitate cross-protection against PCV2d (18). However, the prevalence of PCV2a and PCV2d infections remained high, suggesting the need for the development of a vaccine against PCV2d. Although the prevalence of infections caused by PRRSV and PCV2 was high, exceptionally, viral infections decreased in 2023, whereas bacterial infections, such as HP, PM, and MH, slightly increased (χ^2^ = 11.36, *P* < 0.001).

**Figure 4:**
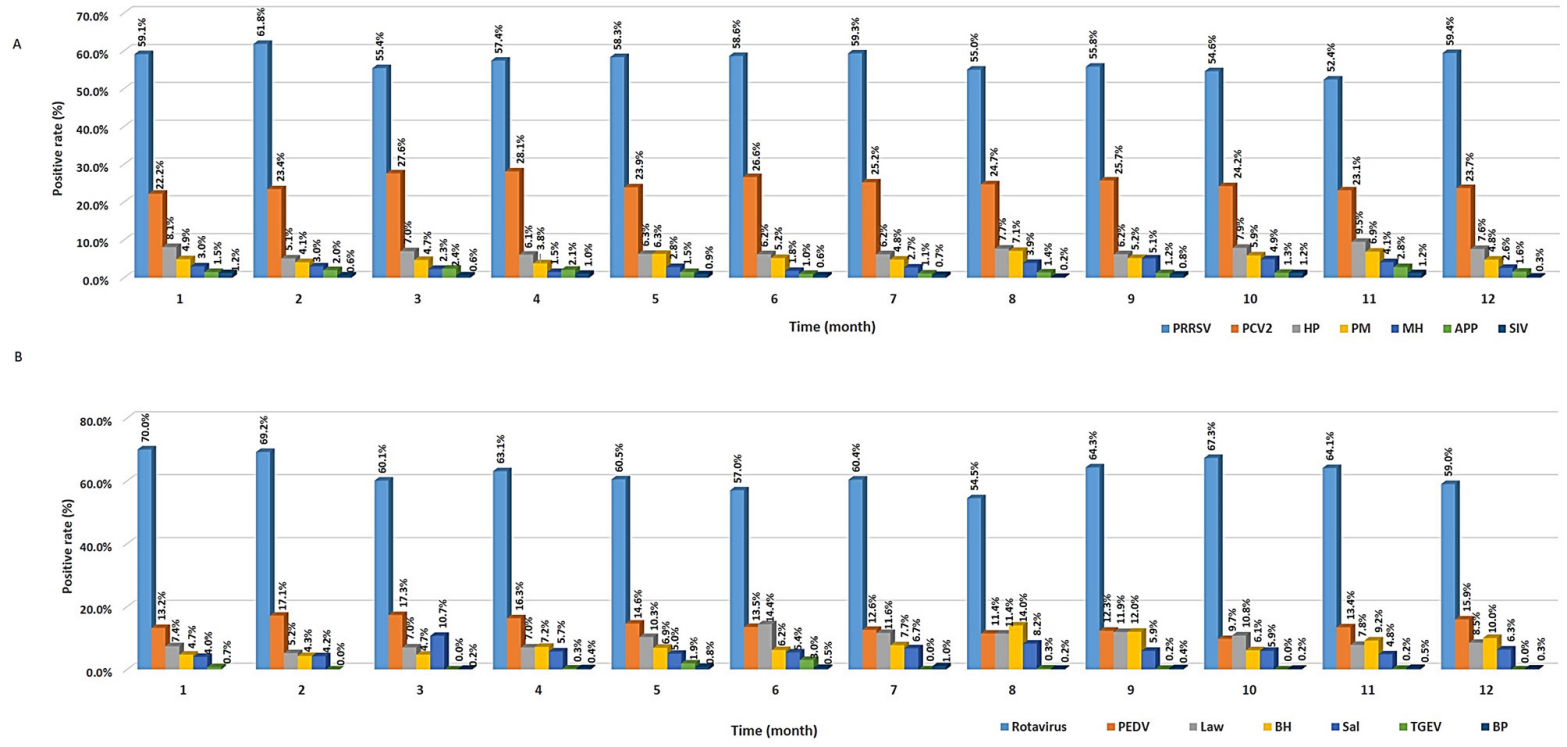
Seasonal prevalence of different viruses and bacteria. **(A)** Respiratory pathogens. **(B)** Digestive pathogens.

To validate the characteristics of seasonal distribution, positive rate of the corresponding pathogens was counted on a monthly basis (Figure 4A). Results showed that the prevalence of PRRSV or HP was slightly higher (χ^2^ = 35.84, *P* < 0.001) in winter (from December to January) than in other seasons, which is consistent with that of a previous study (2). Hence, the prevalence of these diseases was high during winter, as viruses can easily survive in cold environments, while other respiratory pathogens, such as PCV2, appeared similarly regardless of the season.

### Prevalence of digestive pathogens

The overall prevalence of digestive pathogens was 25.3% (*n* = 7,136, 95% CI: 24.7%−25.7%), and the prevalence rates of the viral and bacterial groups were 76.7% (*n* = 5,476, 95% CI: 75.7%−77.7%) and 23.3% (*n* = 1,660, 95% CI: 22.3%−24.3%), respectively. Among the digestive pathogens tested, rotavirus (*n* = 4,430, 62.1%, 95% CI: 60.93%−63.2%) was the most prevalent, followed by PEDV (*n* = 1,009, 14.1%, 95% CI: 13.3%−14.9%), *L. intracellularis* (*n* = 648, 9.1%, 95% CI: 8.4%−9.7%), *B. hyodysenteriae* (*n* = 561, 7.9%, 95% CI: 7.2%−8.5%), *Salmonella* spp. (*n* = 429, 6%, 95% CI: 5.4%−6.5%), TGEV (*n* = 37, 0.5%, 95% CI: 0.3%−0.7%), and *B. pilosicoli* (*n* = 22, 0.3%, 95% CI: 0.2%−0.4%; Figure 2). In this study, the prevalence of rotavirus in South Korea was significantly higher (38.3% vs. 14.1%, χ^2^ = 176.08, *P* < 0.001) than that reported in previous studies (19, 20).

The annual prevalence patterns of rotavirus A (RVA) and rotavirus C (RVC) were investigated individually. Both RVA and RVC were more likely to have decreased since 2021, with the average 3-year prevalence of RVA and RVC was approximately 29.3% (95% CI: 25.6%−33%) and 7.3% (95% CI: 5.1%−9.45%), respectively. RVA and RVC infections are associated with diarrhea and asymptomatic infections in pigs worldwide. Moreover, they have a significant economic impact on pig production (3). Unlike the results of a previous study (7.6% RVA vs. 9.7% RVC), our results showed that infections in RVA were more than four times higher than those in RVC. The co-infection (25.2%, 95% CI: 22.4%−27.9%) between RVA and RVC was also as high as that of RVA infection (Supplementary Table 2). RVA-related diarrhea occurs more often in older and weaning piglets, whereas RVC is more prevalent in young piglets (7). Our results, which showed a high RVA infections, might have been influenced by the samples according to age groups. The prevalence of PEDV was the highest at 17.5% in 2022, with an annual average of 13.8% (95% CI: 9.3%−18.3%), which was lower than that previously reported (>50%) in China (8). The prevalence of TGEV was low at < 1%. The prevalence of viral pathogens that cause diarrhea, such as rotavirus and PEDV, have shown a decreasing trend over the last 3 years. Meanwhile, the prevalence of bacterial pathogens such as *L. intracellularis* and *B. hyodysenteriae* increased in 2023. The economically important *L. intracellurais* was also widespread worldwide and is commonly observed in weaned and growing pigs aged < 4 months (21). The prevalence of *L. intracellularis, B. hyodysenteriae*, and *Salmonella* spp. in our study was lower compared to previously reported studies (19.9%, 10.8%, and 17.7%, respectively). Nevertheless, since these are persistent pathogens (22), there is a need to continuously monitor whether the infections caused by bacterial pathogens is increasing (Figure 3C).

As a result of analyzing the seasonal characteristics of digestive pathogens (Figure 4B), viruses such as rotavirus and PEDV were lowest in August (54.5%, 95% CI: 49.3%−59.6%) and October (9.7%, 95% CI: 7.3%−12.5%) and was highest in January (70%, 95% CI: 66.1%−73.7%) and March (17.3%, 95% CI: 14.6%−20.2%), respectively. The prevalence of bacteria such as *L. intracellularis, B. hyodysenteriae*, and *Salmonella* spp. was highest between June (14.4%, 95% CI: 11.6%−17.6%) and August (14%, 95% CI: 10.6%−17.9% vs. 8.2%, 95% CI: 5.6%−11.4%) and lowest between January (4.7%, 95% CI: 2.5%−5.9%) and February (5.2%, 95% CI: 3.6%−7.2% vs. 4.3%, 95% CI: 2.8%−6.1%), respectively. Unlike that observed for viruses, the reason why the prevalence increases in the summer is because bacterial proliferation becomes more active as the temperature rises. Therefore, environmental and management conditions are essential to control bacterial infections (2). There are potential limitations in this study. Because samples were collected from animals suspected of being infected with pathogens rather than randomly selected, the study population is not representative of the general swine population in Korea. Therefore, further investigation of the prevalence of pathogens in random samples is required.

## Conclusions

The prevalence of respiratory and digestive diseases remained consistent across the years. Porcine reproductive and respiratory syndrome virus (PRRSV), PCV2, rotavirus, HP, PM, and PEDV remain major pathogens threatening the swine farming industry in South Korea. The country is experiencing severe climate change, including shifts in seasonal temperatures and humidity patterns, with a general trend toward warmer temperatures and more frequent extreme weather events. Several respiratory and digestive diseases are occurring due to intensive breeding in large numbers of farms, and co-infections with various diseases complicate differential diagnosis. Recent advances in molecular diagnostic methods, such as multiplex real-time PCR, have enabled the simultaneous and accurate detection of multiple. In addition, to eradicate these disease outbreaks, swine farms and the government are making efforts to identify the outbreak status and causative agent of diseases prevalent in farms. Therefore, farm diseases can be prevented if the disease flow is identified via regular monitoring and proper vaccination and hygiene management programs are implemented. We believe that our study, which includes data on the prevalence of viruses and bacteria, pathogen frequency patterns, annual distribution, and seasonal characteristics, can be helpful in understanding the trend in porcine respiratory and digestive diseases.

## Data Availability

The raw data supporting the conclusions of this article will be made available by the authors, without undue reservation.
