# Supplementary material for: Prevalence of pathogens from clinical samples associated with porcine respiratory and digestive diseases in South Korea from 2021 to 2023
Source: Front Vet Sci. 2025 Jun 30;12:1461935. doi: 10.3389/fvets.2025.1461935 (PMC12258289; doi:10.3389/fvets.2025.1461935)
Supplement: Supplementary file 1 [file Table_1.docx]

**(Supplementary Material) TABLE 1** Commercially available multiplex real-time PCR products used in this study.

|  | Product names | Descriptions |
| --- | --- | --- |
| 1 | Opti HP-PRRSV/PCV2 Multi-qPCR | Highly pathogenic porcine reproductive and respiratory syndrome virus (HP-PRRSV), North American (NA), European (EU),  Porcine circovirus type 2 (PCV2) |
| 2 | Opti SIV/JEV/EMCV Multi-qPCR | Swine influenza virus (SIV), Japanese encephalitis virus (JEV),  Encephalomyocarditis virus (EMCV) |
| 3 | Opti PPV/PCMV/ADV Multi-qPCR | Porcine parvovirus (PPV), Porcine cytomegalovirus (PCMV), Aujezsky disease virus (ADV) |
| 4 | Opti MH/PM/HP/APP Multi-qPCR | *Mycoplasma hyopneumoniae* (MH), *Pasteurella multocida* (PM),  *Haemophilus parasuis* (HP), *Actinobacillus pleuropneumoniae* (APP) |
| 5 | Opti PEDV/TGEV/RVA/RVC Multi-qPCR | Porcine epidemic diarrhea virus (PEDV), Transmissible gastroenteritis virus (TGEV), Rotavirus A, Rotavirus C |
| 6 | Opti SLBB Multi-qPCR | *Salmonella* spp., *Lawsonia intracellularis,*  *Brachyspira hyodysenteriae, Brachyspira pilosicoli* |
