# Supplementary material for: Prevalence of pathogens from clinical samples associated with porcine respiratory and digestive diseases in South Korea from 2021 to 2023
Source: Front Vet Sci. 2025 Jun 30;12:1461935. doi: 10.3389/fvets.2025.1461935 (PMC12258289; doi:10.3389/fvets.2025.1461935)
Supplement: Supplementary file 2 [file Table_2.docx]

**(Supplementary Material) TABLE 2** Number of co-infected pathogens between 2021 and 2023.

| Co-infected pathogens | Year, n (%) | | | |
| --- | --- | --- | --- | --- |
|  | 2021 | 2022 | 2023 | Total |
| PRRSV + PCV2 | 607 (15.3) | 700 (17.8) | 706 (17.2) | 2,013 (16.7) |
| PRRSV-1 + PRRSV-2 | 628 (9.3) | 419 (6) | 411 (5.6) | 1,458 (7) |
| RVA + RVC | 552 (24.7) | 833 (27.8) | 437 (23) | 1,822 (25.2) |
